# The functional roles, cross-talk and clinical implications of m6A modification and circRNA in hepatocellular carcinoma

**DOI:** 10.7150/ijbs.62767

**Published:** 2021-07-22

**Authors:** Sha Qin, Yitao Mao, Xue Chen, Juxiong Xiao, Yan Qin, Luqing Zhao

**Affiliations:** 1 Department of Pathology, Xiangya Hospital, Central South University, Changsha, Hunan, China; and Department of Pathology, School of Basic Medical Science, Xiangya School of Medicine, Central South University, Changsha, Hunan, China.; 2 Department of Radiology, Xiangya Hospital, Central South University, Changsha, Hunan, China.; 3 National Clinical Research Center for Geriatric Disorders, Xiangya Hospital, Central South University, Changsha, Hunan, China.; 4 Early Clinical Trial Center, Hunan Cancer Hospital and The Affiliated Cancer Hospital of Xiangya School of Medicine, Central South University, Changsha, Hunan, China.

**Keywords:** N6-methyladenosine (m6A) modification, circRNA, Hepatocellular carcinoma (HCC), Biomarkers, Therapeutic targets

## Abstract

Hepatocellular carcinoma (HCC) is one of the leading causes of cancer-related deaths worldwide. HCC has high rates of death and recurrence, as well as very low survival rates. N6-methyladenosine (m6A) is the most abundant modification in eukaryotic RNAs, and circRNAs are a class of circular noncoding RNAs that are generated by back-splicing and they modulate multiple functions in a variety of cellular processes. Although the carcinogenesis of HCC is complex, emerging evidence has indicated that m6A modification and circRNA play vital roles in HCC development and progression. However, the underlying mechanisms governing HCC, their cross-talk, and clinical implications have not been fully elucidated. Therefore, in this paper, we elucidated the biological functions and molecular mechanisms of m6A modification in the carcinogenesis of HCC by illustrating three different regulatory factors ("writer", "eraser", and "reader") of the m6A modification process. Additionally, we dissected the functional roles of circRNAs in various malignant behaviors of HCC, thereby contributing to HCC initiation, progression and relapse. Furthermore, we demonstrated the cross-talk and interplay between m6A modification and circRNA by revealing the effects of the collaboration of circRNA and m6A modification on HCC progression. Finally, we proposed the clinical potential and implications of m6A modifiers and circRNAs as diagnostic biomarkers and therapeutic targets for HCC diagnosis, treatment and prognosis evaluation.

## Introduction

**Figure 1:**
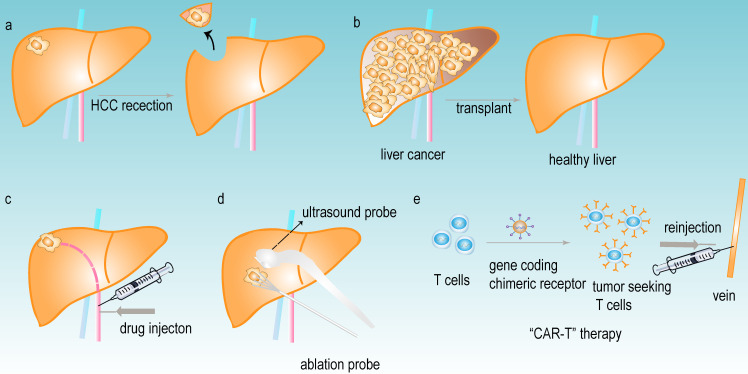
** The common treatments of HCC.** (a) HCC resection. (b) liver transplant. (c) trans-arterial chemoembolization (TACE). (d) percutaneous ablation. (e) chimeric antigen receptor-engineered T-cell therapy (CAR-T).

Primary liver cancer is one of the most common malignancies, which has a low survival rate. It is currently the sixth most commonly diagnosed cancer and the third leading cause of cancer mortality worldwide, and the hepatocellular carcinoma (HCC) comprises 75%-85% of primary liver cancer 1. With recognition of the severity and danger of HCC, pharmacological and nonpharmacological treatments have achieved great progress in the therapeutic field of HCC. The nonpharmacological treatments mainly include HCC resection (**Fig 1a**) 2, liver transplantation (**Fig 1b**) 3, trans-arterial chemoembolization (TACE) (**Fig 1c**) 4, percutaneous ablation (**Fig 1d**) 5 and chimeric antigen receptor engineered T-cell immunotherapy (CAR-T) (**Fig 1e**) 6. The pharmacological treatments largely consist of small molecule targeted drug therapies, such as sorafenib 7, lenvatinib, regrofenib, and monoclonal antibodies such as nivolumab 8. These drugs are used mainly for the systemic treatment of advanced HCC. However, most patients are diagnosed at advanced stages due to the lack of obvious early symptoms and limitations in early detection or screening. Despite the variety of treatments for HCC, the overall survival rate of patients is still far from satisfactory; therefore, developing more effective early diagnosis and treatment methods for HCC patients is critical.

N6-methyladenosine (m6A) has been shown to be the most abundant modification in eukaryotic RNAs and plays an essential role in a variety of biological processes, including RNA stability, translation, splicing, degradation, and export 9, 10. An increasing number of studies have proven that aberrant regulation of m6A modification is associated with a wide variety of human malignancies, including breast cancer, lung cancer, colorectal cancer, and HCC 11, 12.

circRNA is a circular noncoding RNA formed by back-splicing. Due to its covalent closed-loop structure, circRNA can prevent degradation from RNA exonuclease; thus, circRNA is more stable than linear mRNA. Accumulating evidence has indicated that the functions of circRNAs in gene regulation are closely related to the development of HCC and that circRNAs might be promising potential biomarkers and therapeutic targets for the diagnosis and treatment of HCC 13.

In this paper, we elucidated the functional roles, cross-talk, and underlying interplay mechanisms between m6A modification and circRNA in HCC carcinogenesis and development. And we demonstrated the clinical implications of m6A modifiers and circRNAs in the diagnosis and treatment of HCC as promising diagnostic biomarkers and therapeutic targets. In particular, we also briefly identified common databases and research methods for investigating the roles and interactions of m6A and circRNA, which could provide novel in-depth research insights and avenues for further studies on the functions and mechanisms of m6A and circRNAs in HCC.

## The roles of m6A modification in HCC

### The components of m6A modification

**Figure 2:**
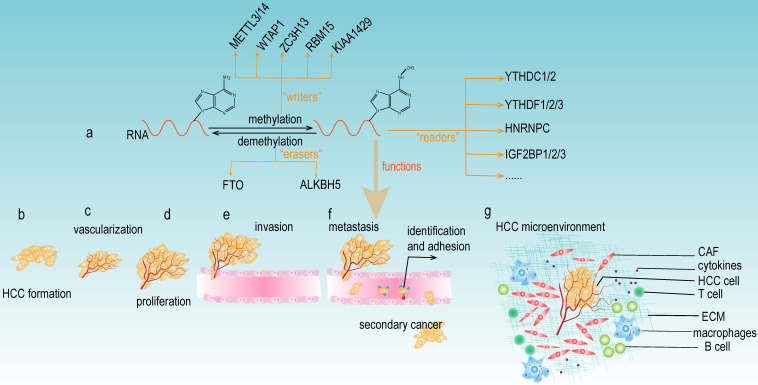
** The functions of m6A modification in HCC.** (a) The components of m6A modification. m6A “writers” catalyze the m6A modification of adenosine on RNA and mainly include METTL3/14, WTAP, ZC3H13, RBM15, and KIAA1492. Removing the methylation of RNA needs the functions of m6A “erasers” that mainly consist of FTO and ALKBH5. m6A “readers” (such as YTHDC1/2, YTHDF1/2/3, HNRNPC, and IGF2BP1/2/3) recognize m6A modification sites and exert various biological functions. (b-g) Multiple functions of m6A modification in HCC development. m6A modifiers could affect the tumor formation, vascularization, proliferation, invasion, metastasis, and the microenvironment of HCC.

m6A modification consists of the following three main components: m6A "writers", "erasers", and "readers" (**Fig 2a**). The "writers" are methyltransferases that function to promote the methylation modification of RNA. They mainly include METTL3, METTL14, WTAP1, RBM15, and KIAA1429 14. The "erasers" are the enzymes that promote the demethylation of m6A modification. Currently, the "erasers" mainly include FTO and ALKBH5. The presence of these enzymes indicates that m6A modification is a dynamic and reversible process. Notably, m6A modification relies on “readers” to perform its biological functions. The "readers" selectively recognize and bind the m6A modification site on the RNA and subsequently change the RNA structures, thus leading to different functions in RNA biology. The YTH protein domain families are the major m6A “readers”, which consist of YTHDC1 (in the nucleus), YTHDC2, YTHDF1, YTHDF2 and YTHDF3 (in the cytoplasm). In addition, the heterogeneous nuclear ribonucleoprotein (HNRNP) families (including HNRNPA2B1, HNRNPC and HNRNPG), NF-κβ-associated protein (NKAP) and IGF2BP families are also m6A "readers" that exert important roles in various biological functions of m6A modification.

### The m6A “writers” and HCC

#### METTL3 and HCC

METTL3 has a catalytic subunit that can bind S-adenosylmethionine (SAM) (which provides the methyl group) and catalyze methylation 15, and METTL3 was the first identified methyltransferase catalyzing m6A modifications of mRNA and noncoding RNA 16. Transcriptome sequencing revealed that METTL3 was significantly upregulated in HCC tissues. Knockdown of METTL3 significantly reduced the proliferation, migration and colony formation capacities of HCC cells. Overexpression of METTL3 was associated with poor prognosis in HCC patients 17. Additionally, a gene set enrichment analysis of HCC specimens demonstrated that METTL3 expression was closely related to glycolysis and the mTOR signaling pathway. Overexpression of METTL3 could activate the mTOR complex 1 (mTORC1) and facilitate glycolysis in HCC cells through mTORC1 pathway 18. Moreover, another study discovered that the Hippo pathway was involved in the METTL3-mediated formation of vasculogenic mimicry and this m6A modification could affect the translation of YAP1 mRNA, thus promoting the angiogenesis of HCC 19. Knockdown of METTL3 expression under hypoxic conditions was also reported to be able to promote the expression of angiogenic genes and activate autophagy-related pathways in HCC cells. Furthermore, METTL3 deletion abolished METTL3-mediated regulation of FOXO3 mRNA stability and markedly enhanced resistance to sorafenib treatment in HCC, therefore uncovering the critical functions of METTL3-mediated m6A modification in the hypoxic microenvironment of HCC 20.

### METTL14 and HCC

METTL14 and METTL3 share 43% homology in their molecular structure 21. As a homolog of METTL3, the downregulation of METTL14 was closely related to tumor metastasis and it acted as an adverse prognostic factor of recurrence-free survival in HCC patients. METTL14 could interact with the microprocessor protein DGCR8 and positively regulated pri-miR-126 biogenesis in an m6A-dependent manner 22. METTL14 could also inhibit the metastasis of HCC by upregulating the m6A methylation of CSAD, GOT2, and SOCS2 23. Another report indicated that METTL14 modulated the EGFR/PI3K/AKT signaling pathway to impair the migration, invasion and epithelial-mesenchymal transition (EMT) of HCC cells 24. Additionally, hepatocyte nuclear factor 3γ (HNF3γ) expression was significantly downregulated in HCC patients and was negatively correlated with HCC progression and survival rate. The reduction in HNF3γ expression was mediated by METTL14-dependent m6A methylation of HNF3γ mRNA. In particular, overexpression of HNF3γ could reduce the drug resistance of HCC by upregulating the expression of OATP1B1 and OATP1B3, which are the two major membrane transport proteins affecting sorafenib absorption, thus sensitizing HCC cells to sorafenib-induced growth repression and apoptosis 25.

### Other “writers” and HCC

In addition to two major m6A "writers", METTL3 and METTL14, several other "writers" have also been discovered to govern HCC development and progression. For example, KIAA1429 was remarkably upregulated in HCC tissues and was strongly associated with the proliferation and metastasis of HCC cells. KIAA1429 induced m6A methylation on the 3'-untranslated region (3'-UTR) of GATA3 mRNA and led to the disassociation of the RNA-binding protein (RBP) HuR, thus resulting in the degradation of GATA3 mRNA and promoting the formation of HCC 26. WTAP was also highly expressed in HCC and was an independent predictor of HCC patient survival. WTAP boosted HCC cell proliferation and accelerated HCC progression via the HuR-ETS1-p21/p27 axis 27. Accumulating evidence has demonstrated that multiple growth factors can stimulate HCC cell proliferation by activating the Ras/Raf-1/ERK pathway, which is essential for the tumorigenesis of HCC 28. Recently, bioinformatics analysis revealed that ZC3H13 and KIAA1429 could be practical and reliable prognostic indicators of HCC 29; however, the molecular mechanisms of ZC3H13 in the RAS-ERK pathway and their interactions in HCC have not been fully elucidated, therefore further researches are needed to be conducted in the future.

### The m6A “erasers” and HCC

#### FTO and HCC

Fat mass and obesity-associated protein (FTO) is well known for its pivotal roles in the regulation of fat mass, adipogenesis and body weight. FTO mRNA levels were reported to be significantly lower in HCC tissues. The expression of FTO was reduced in the HCC cells and was closely associated with AFP levels, tumor size, metastasis and vascular invasion 30, 31. Another study demonstrated that overexpression of FTO was associated with poor prognosis in HCC patients. Knockdown of FTO suppressed the proliferation of HCC and induced G0/G1 phase arrest. Mechanistically, FTO resulted in the demethylation of PKM2 mRNA and accelerated its translation process, thus facilitating HCC malignant progression 32. Moreover, SIRT1 exerted an oncogenic role by downregulating the expression of FTO. Guanine nucleotide-binding protein G (o) subunit alpha (GNAO1) was identified as the downstream target of FTO and a tumor suppressor in HCC. Depletion of FTO by SIRT1 could improve the m6A modification of GNAO1 and downregulated its mRNA expression level, therefore contributing to HCC metastasis 33. Additionally; FTO is indispensable for the regulation of energy homeostasis and glucose metabolism in HCC. Furthermore, FTO acted as a protector in HCC carcinogenesis, and FTO-dependent dynamic mRNA demethylation of CUL4A exerted critical functions in the initiation and development of HCC 34.

### ALKBH5 and HCC

ALKBH5 expression in HCC tissues was significantly lower than that in normal tissues, and the absence of ALKBH5 predicted poor overall survival and disease-free survival in HCC patients 30, 35. ALKBH5-mediated m6A demethylation resulted in the posttranscriptional repression of LY6/PLAUR domain 1 (LYPD1), which could be stabilized by the m6A “reader” IGF2BP1. In addition, LYPD1 was identified to induce the oncogenic behaviors of HCC. These results suggested that the dysregulation of the ALKBH5/LYPD1 axis could accelerate the progression and invasiveness of HCC.

### The m6A “readers” and HCC

Many m6A “readers” such as YTHDC1/2, YTHDF1/2/3, HNRNPA2B1, HNRNPC, HNRNPG, NKAP and IGF2BP1/2/3 have been reported to play essential roles in HCC development. For example, Liu et al. revealed that YTHDC2 could act as a promising target for specific therapeutic strategy in unresectable HCC patients 36. Li et al. found that the IGF2BP1/IGF2BP3 could stabilize LINC01138 transcript and was associated with the malignant features and poor prognosis of HCC patients 37. In addition, the overexpression of IGF2BP2 enhanced the stability of FEN1 mRNA, so as to promote HCC proliferation 38. HNRNPA2B1 was also involved in HCC metastasis. Wang et al. showed that HNRNPA2B1decreased the stability of p52 and p65 mRNA. Besides, miR503HG could promote the degradation of HNRNPA2B1 and inhibit HCC migration through suppressing the NF-κB signaling pathway 39. NKAP was highly expressed in HCC tissues. And NKAP promoted the proliferation and invasion in HCC cell through AKT signaling pathway 40. Zhao et al. discovered that YTHDF1 was significantly upregulated in HCC and positively related to pathological stage. Kaplan-Meier analysis suggested that lower YTHDF1 expression levels were associated with better survival in HCC patients. Moreover, YTHDF1 acted as a key regulator of cell cycle progression and metabolism in HCC, and it became a potential novel therapeutic and prognostic target for HCC 41. Additionally, bioinformatics analysis predicted that YTHDF1 and YTHDF2 were significantly positively correlated with overall survival, WHO stage, and pathological grade 42, 43. Zhang et al. demonstrated that YTHDF2 expression was negatively associated with survival time in HCC. In addition, YTHDF2 expression was positively related to OCT4 expression and m6A levels in the 5'-UTR of OCT4 mRNA in HCC clinical specimens. Therefore, YTHDF2 facilitated the HCC stemness phenotype and tumor metastasis by regulating m6A methylation of OCT4 44, 45. Another study identified that the downregulation of YTHDF2 was specifically induced by hypoxia in HCC cells, and overexpression of YTHDF2 could inhibit cell proliferation and tumor growth in HCC cells. Mechanistically, YTHDF2 directly bound the m6A modification site in the EGFR 3'-UTR to accelerate the degradation of EGFR mRNA. This was the first report indicating that YTHDF2 might act as a tumor suppressor to suppress cell proliferation and growth by destabilizing EGFR mRNA in HCC development 46.

As mentioned above, the "writers", "erasers" and "readers" of m6A modification are significantly involved in the development of HCC (**Table 1**), and this modification can either facilitate or suppress the progression of HCC. These paradoxical results caused by m6A modification may be attributable to different downstream targets of m6A, which could impact diverse signaling pathways and trigger different biological functions. Further studies on the specific regulatory mechanisms are still needed. m6A regulators have been reported to be involved in various aspects of HCC carcinogenesis, including tumor formation, vascularization, proliferation, invasion, metastasis, and the tumor microenvironment (**Fig 2b-g**). Meanwhile, most current research investigating m6A regulators focused on the downstream molecular mechanisms of specific genes; however, there are relatively insufficient studies exploring the upstream modulatory mechanisms, thus providing new avenues and directions to conduct in-depth research concerning m6A modification and HCC development.

## The biogenesis and functions of circRNA

Accumulating evidence has demonstrated that a large number of circRNAs are aberrantly expressed in HCC tissues, suggesting that these circRNAs may play a vital role in the development of HCC. Based on their different origins, circRNAs can be divided into the following three groups: exonic circRNAs (ecRNAs), exon-intron circRNAs (eIciRNAs) and intronic circRNAs (ciRNAs). Exonic circRNAs are generated mainly from the exons of protein-coding genes 47. When exons are cyclized by introns and exons, they generated eIciRNAs. eIciRNAs are largely localized in the nucleus and interact with U1 snRNP to promote the transcription of their parental genes 48. Due to the debranching failure, ciRNA can accumulate in large quantities at their transcription sites. ciRNAs are associated with the elongation of Pol II and act as positive regulators of Pol II transcription, thus exerting cis-regulatory effects on their parental coding genes 49.

### The biogenesis of circRNA

**Figure 3:**
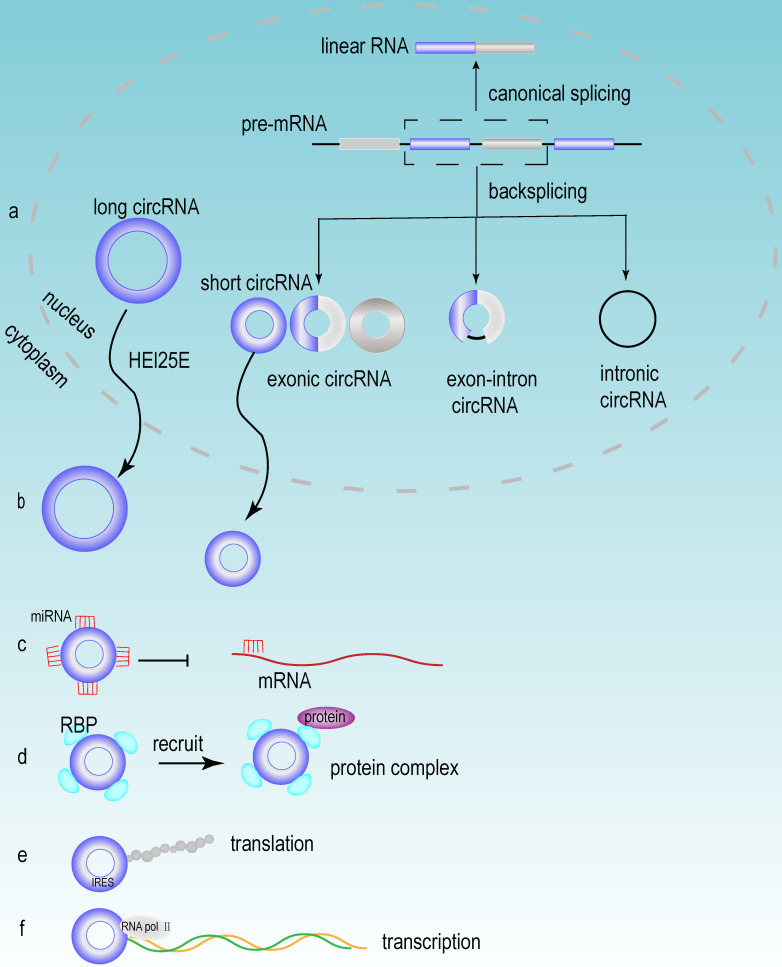
** The biogenesis and functions of circRNA.** (a) The mechanisms of circRNA biogenesis. The formation of circRNA was owing to back-splicing. There were three types of circRNA named exon circRNA, exon-intronic circRNA and intronic circRNA, which were generated through different splicing methods. (b) Nuclear export of circRNA. Most circRNAs executed their first functions through exporting the nucleus circRNA to the cytoplasm, and this process mainly depended on the length of circRNA. (c) circRNA acted as miRNA sponge. (d) circRNA interacted with RBP. (e) circRNA could be translated in a cap-independent manner via the internal ribosome entry site (IRES). (f) circRNA regulated transcription.

circRNA is an abundant, stable, conserved, nonrandom product generated by RNA splicing that may participate in the regulation of gene expression 50. Typically, eukaryotic pre-mRNA splicing is catalyzed by a spliceosomal mechanism that removes introns and attaches exons, resulting in the formation of linear RNA transcripts. The generation of circRNA relies on a back-splicing mechanism that backlinks the downstream splice donor site to the upstream splice acceptor site and forms a covalently closed circular RNA transcript and a selectively spliced linear RNA that skips exons (**Fig 3a**) 51. Competition exists between the back splicing of circRNA and typical splicing of linear RNA, and this competition is modulated by cis- and trans- elements 52. Base pairing between highly supplementary transposable elements can boost back splicing 53. The loop formation of intron sequences on the flank of the downstream splice donor site and the upstream splice acceptor site keeps these sites in close proximity, which may be the main foundation for the back splicing of circRNA. This loop formation can be achieved by base pairing between reverse repeat elements (e.g. Alu elements) located in the upstream and downstream introns 54. The efficiency of exon loop formation can be regulated by competition between RNA pairs. The alternate formation of reverse repeat Alu elements and the competition between them results in multiple circRNA transcripts from a single gene 55. The biogenesis of circRNA is regulated by various factors, including intron sequences, enzymes and protein factors 56. Adenosine deaminase ADAR1 is a type of RNA editing enzyme. ADAR1 has been reported to negatively regulate circRNA expression, and the knockdown of ADAR1 induces the elevation of circRNA expression 57. RBPs may play an activating or inhibiting role in circRNA formation by regulating variable splicing factor Quaking (QKI) levels. And QKI binding motifs of introns exert an essential impact on circRNA abundance 58.

### The general functions of circRNA

#### Nucleus export process of circRNA

After the formation of circRNAs, most of circRNAs (except for ciRNAs) are exported to the cytoplasm to perform different biological functions 59. Research has revealed that the process of circRNA exporting the nucleus is closely related to its own length 60, 61. circRNA is transported from the nucleus to the cytoplasm via the ATP-dependent RNA helicase DDX39A (also known as URH49) and the spliceosomal RNA helicase DDX39B (also known as UAP56). And this mode of transport is closely related to the length of circRNA. When the circRNA length is longer than 800 bases, the nuclear export process relies on Hel25E. With the transcript length increasing, the export of circRNA becomes increasingly dependent on Hel25E (**Fig 3b**) 62.

### circRNA acts as a miRNA sponge

miRNA is an endogenous short-stranded noncoding RNA that consists of approximately 20-25 nucleotides. Current studies have shown that miRNAs can specifically bind to mRNA and regulate gene expression through posttranscriptional regulation, thus contributing to mRNA silencing or degradation 63. circRNAs can function as miRNA sponges and can compete for mRNA-miRNA binding sites, thereby inhibiting mRNA suppression (**Fig 3c**). When circRNA functions as a miRNA sponge, the stoichiometric relationship between the miRNA binding site of circRNA and the mRNA binding site of miRNA is an important factor to influence the efficiency of circRNA function 64. Notably, circRNAs can act as sponges for multiple different miRNAs, therefore, the same circRNA can affect multiple different miRNAs and perform different biological functions 65. Different circRNAs have different functions, but some similar biological functions can be performed by different circRNAs. For example, circRNA_0016624 could act as a molecular sponge for miR-98 and enhanced the expression of BMP2; therefore, circRNA_0016624 could prevent osteoporosis 66. circRNA_33186 acted as a molecular sponge to directly bind and repress miR-127-5p, thereby increasing the expression of MMP-13, which is involved in the pathogenesis of osteoarthritis 67. In glioblastoma, circNT5E acted as a sponge for miR-422a and could inhibit the activity of miR-422a, thereby promoting tumor cell proliferation, migration and invasion 68.

### The translation capacity of circRNA

Although circRNA is a circular noncoding RNA, several studies in recent years have shown that it also has translation capacity 69. In recent years, several circRNAs have been discovered to have translational functions, which have biological roles in human diseases. The discovery of these circRNAs and their encoded peptide-enriched genomes helped us to study the etiology of diseases and facilitated the development of biotechnology 70. circMYBL2 has been proven to be translated, and it could regulate FLT3 kinase levels so as to specifically affect acute leukemia that caused by internal tandem repeat mutations of FLT3. The mechanism of this function is that circMYBL2 enhances the translation efficiency of FLT3 kinase by increasing the binding of polypyrimidine binding protein 1 (PTBP1) to the mRNA of FMS-like tyrosine kinase-3 (FLT3) 71. Furthermore, although circRNAs lack essential components such as the 5' cap and poly(A) tail for cap-dependent translation, circRNAs can still be translated in a cap-independent manner via the internal ribosome entry site (IRES) (**Fig 3e**) 72.

### Other functions of circRNA

circRNAs can also regulate gene expression at the transcriptional level (**Fig 3f**). For example, eIciRNAs, which are mainly localized in the nucleus, interact with U1 snRNP to promote the transcription of their parental genes 48. circRNA also participates in regulating the variable splicing of transcripts, and researchers have proposed a mechanism by which circRNA can regulate transcription through cyclization, i.e., the formation of circRNA can compete with splicing of linear RNAs for common pre-mRNAs, thereby affecting the abundance of linear RNAs 52. In addition, circRNAs can also interact with RBPs (**Fig 3d**) 73. In detail, circRNAs participate in epigenetic regulation and posttranslational regulation of proteins through the following methods. They could act as protein sponges and enhance protein function. They could also act as scaffolds to mediate some complex formation between specific enzymes and substrates. Additionally, they recruit proteins to specific sites to regulate their activity or influence their expression 54. The interaction of circRNA with RBP is considered to be an important function of circRNA and is the basic mechanism for circRNA to exert functions, including circRNA generation, translation, transcriptional regulation of target genes and extracellular transport 74.

## The roles of circRNA in HCC

### circRNA and HCC proliferation and apoptosis

circRNA_0001955 increased TRAF6 and MAPK11 expression through adsorption of miR-516a-5p and further promoted the tumorigenesis of HCC 75. circRHOT1 promoted HCC cell growth of HCC by increasing the level of NR2F6 and worsened the prognosis of HCC patients 76. Knockdown of circRNA_0067934 not only significantly inhibited the proliferation of Hep3B and HuH7 cells, but also induced the apoptosis of HCC cells though the FZD5/Wnt/β-catenin signaling pathway 77. circRNA_0000502 promoted the malignant progression of HCC by downregulating the expression of miR-124. Knockdown of circRNA_0000502 significantly reduced the proliferation ability of HCC cells and increased cell apoptosis 78. hsa_circ_0051443 was delivered from normal cells to HCC cells via exosomes, and it can facilitate apoptosis and block the cell cycle to repress malignant behaviors of HCC by regulating extracellular communication in the tumor microenvironment (TME) 79.

### circRNA and HCC invasion and metastasis

Downregulation of circRNA cSMARCA5 (hsa_circ_0001445) in HCC was significantly associated with invasion and was an independent risk factor for overall survival and recurrence-free survival after hepatectomy. cSMARCA5 was identified to promote the expression of the tumor suppressor TIMP3 by acting as a miR-17-3p and miR-181b-5p sponge, thereby inhibiting the migration of HCC cells 80. circASAP1 (a circRNA from exons 2 and 3 of the ASAP1 gene, hsa_circ_0085616) was identified by circRNA sequencing to be associated with metastasis in the lungs of HCC patients after radical resection. circASAP1 was overexpressed in HCC with high metastatic potential. circASAP1 promotes HCC invasion through the modulation of the miR-326/miR-532-5p-MAPK1 signaling pathway and mediation of HCC-associated macrophage infiltration through the regulation of the miR-326/miR-532-5p-CSF-1 pathway 81. circ-ADD3 negatively correlated with vascular invasion, intrahepatic metastasis and distant metastasis. circ-ADD3 (hsa_circ_0020007) downregulated the stability of EZH2 and increased the expression of antimetastatic genes to suppress metastasis in HCC 82. In addition, circMTO1 (hsa_circRNA_0007874/hsa_circRNA_104135) was a circRNA that was downregulated in HCC tissues and promoted HCC invasion by acting as a sponge of oncogenic miR-9 to promote p21 expression 83. circRNA_0003998 contributed to EMT in HCC via the circRNA_0003998/miR-143-3p/FOSL2 axis and circ_0003998/PCBP1/CD44V6 axis. circRNA_0003998 might act as a ceRNA that alleviated the suppressive effect of miR-143-3p on FOSL2 (a stimulator of EMT), while circRNA_0003998 could bind to PCBP1 and increase the expression level of CD44V6 (a gene associated with EMT) 84. In addition, overexpression of circRNA Cdr1 (hsa_circ_0001946) accelerated the migration of HCC cells. Cdr1as could promote the expression of AFP through adsorption of miR-1270. Additionally, circRNA Cdr1as exosomes from HCC cells accelerated the migration of surrounding normal cells 85. Another survey concluded that the expression of circRNA_104718 accelerated cell migration and invasion, while inhibited the apoptosis of HCC cells through the microRNA-218-5p/TXNDC5 signaling pathway 86. In contrast, overexpression of exosomal circRNA_100338 significantly enhanced the invasive ability of HCC cells, so overexpression of exosomal circRNA_100338 could promote metastasis by enhancing invasiveness and angiogenesis, and persistent high expression of exosomal circRNA_100338 may be a risk indicator for metastasis and poor survival of HCC 87. In addition, circRNA_5692 expression was demonstrated to be downregulated in HCC tissues, and overexpression of circRNA_5692 attenuated malignant behavior by reducing miR-328-5p expression and enhancing DAB2IP expression. This study proved that circRNA inhibited the invasion and metastasis of HCC by regulating the circRNA-5692-miR-328-5p-DAB2IP pathway. These findings may provide potential new targets for the diagnosis and treatment of HCC 88.

### circRNA and HCC angiogenesis

Angiogenesis is an extremely vital mechanism for tumors to eliminate carbon dioxide and metabolic waste and to acquire adequate nutritional support 89. Vasculogenesis mimicry (VM), a novel pattern of vascularization, is involved in HCC progression and metastasis. Androgen receptor (AR) suppressed the formation of HCC VM by downregulating circRNA7/miRNA7-5p/VE-cadherin/Notch4 signaling axis in HCC, which may be novel therapies against HCC 90. circGFRA1 was aberrantly highly expressed in HCC tissues and cells, which may lead to poor prognosis. Knockdown of circGFRA1 suppressed angiogenesis and caused inhibition of migration in HCC cells by binding to miR-149 91. hsa_circ_0000092 competitively bound to miR-338-3p and thereby upregulating the expression of HN1. And the circ_0000092 deletion or miR-338-3p elevation repressed HCC cell proliferation, migration, invasion and angiogenesis 92. As a critical component of the TME, endothelial cells were involved in angiogenesis process and could affect the development of HCC. circ_4911 and circ_4302 were downregulated in HCC, and overexpression of circ_4911 and circ_4302 inhibited the proliferation and migration of human umbilical vein endothelial cells (HUVECs), thereby restraining the angiogenesis of tumors 93.

### circRNA and HCC microenvironment

circRNAs are well established to recruit and reconstitute key components in the tumor microenvironment (TME) and to mediate various signaling pathways that involving tumorigenesis, angiogenesis, immune response, metabolism reprogramming and metastatic progression. circRNA interacts with critical cellular components of the TME (such as cancer-associated endothelial cells (CAEs), immune cells, cancer-associated fibroblasts (CAFs) and cancer stem cells) and noncellular components (cytokines, growth factors, metabolites, and extracellular matrix (ECM)) 94. Combining targeting circRNA and coactivating components in the TME may achieve superior therapeutic efficacy and become a new pattern of future tumor treatment 95. circRNA affects the TME from multiple perspectives, including immune monitoring, angiogenesis, hypoxia, stromal remodeling and the resistance of radiation treatments 96. In HCC, circMET (hsa_circ_0082002) promoted the EMT process, and it enhanced the immunosuppression of HCC through the Snail/DPP4/CXCL10 axis 97. Additionally, circUHRF1 caused immunosuppression through inducing NK cell dysfunction in HCC. circUHRF1 inhibited the secretion of NK cell-derived IFN-γ and TNF-α and it was associated with a decreased NK cell infiltration in TME 98. circMAT2B (hsa_circ_0074854) expression was upregulated in HCC, which shortened the overall survival of patients. Mechanistically, circMAT2B promoted glycolysis and accelerated HCC progression by acting as a miR-338-3p sponge and upregulating the expression level of the miR-338-3p target gene PKM2, which encoded a key enzyme in the glycolysis process 99.

### circRNA and HCC drug resistance

circRNA_101505 was diminished in HCC cisplatin-resistant tissues and cell lines, which indicated poorer survival outcomes. circRNA_101505 could sensitize HCC cells to cisplatin by acting as a sponge for miR-103, thereby stimulating the expression of oxidored-nitro domain-containing protein 1 (NOR1), which suppressed the growth of cancer cells and enhanced cisplatin toxicity 100. In addition, the expression of circRNA-SORE (circRNA_104797) was upregulated in sorafenib-resistant HCC cells. circRNA-SORE bound to the oncogenic protein YBX1 in the cytoplasm and prevented the interaction of YBX1 with the E3 ubiquitin ligase PRP19, thereby blocking PRP19-mediated degradation of YBX1. Therefore, the deletion of circRNA-SORE could significantly enhance the cell killing ability of sorafenib 59. In HCC, circRNA_102272 expression was upregulated, which facilitated cisplatin resistance in HCC through regulation of the miR-326/RUNX2 axis 101. The expression of circRNA_101237 may increase DNA repair, increase drug efflux, and alter cell accumulation so as to promote cisplatin resistance in HCC, which is also associated with tumor size, lymph node metastasis and TNM stage. Therefore circRNA_101237 was an independent predictor of prognosis in HCC patients 102. circFN1 (hsa_circ_0058124) was found to be able to promote the expression of E2F1 by acting as a miR-1205 sponge; thus, inhibition of circFN1 could enhance the sensitivity of HCC cells to sorafenib 103. circARNT2 (hsa_circ_0104670) regulated cisplatin resistance through the miR-155-5p/PDK1 axis, while downregulation of circARNT2 inhibited cell proliferation and enhanced the sensitivity of HCC cells to cisplatin 104. Knockdown of circ_0003998 inhibited drug-resistant cell viability, migration, invasion and EMT, and also enhanced the toxicity of doxorubicin to HCC cells. circ_0003998 acted as a ceRNA for miR-218-5p to regulate the expression of EIF5A2, and reduced sensitivity to doxorubicin in HCC cells by regulating the miR-218-5p/EIF5A2 axis 105. Knockout of circ_0003418 stimulated the proliferation, migration and invasion of HCC cells. circ_0003418 enhanced the sensitivity of HCC cells to cisplatin by inhibiting the Wnt/β-catenin signaling pathway 106.

In conclusion, circRNA is generated by back splicing and lacks a cap and tail structure. It is also less susceptible to degradation by RNA enzymes and is more stable than linear RNA. The process of its generation is regulated by multiple factors, including intron sequences, enzymes and protein factors. Most circRNAs function in the cytoplasm, and the process of circRNAs export from the nucleus depend on their own length. circRNAs usually act as miRNA sponges to exert their biological functions. Certain circRNAs have cap-independent translation potential. circRNAs play significant roles in HCC carcinogenesis (**Table 2**). Most circRNAs act as miRNA sponges and inhibit the suppression of target mRNAs to affect the malignant behaviors of HCC, including cell growth, proliferation, cell cycle, apoptosis, invasion, metastasis, angiogenesis, tumor microenvironment remodeling, immune imbalance, metabolism reprogramming, and drug resistance. In addition, some circRNAs can directly affect signaling pathways to regulate the progression of HCC. The detailed regulatory mechanisms and interactions are worthwhile to conduct further investigations.

## Cross-talk and interplay between m6A modification and circRNA in HCC

**Figure 4:**
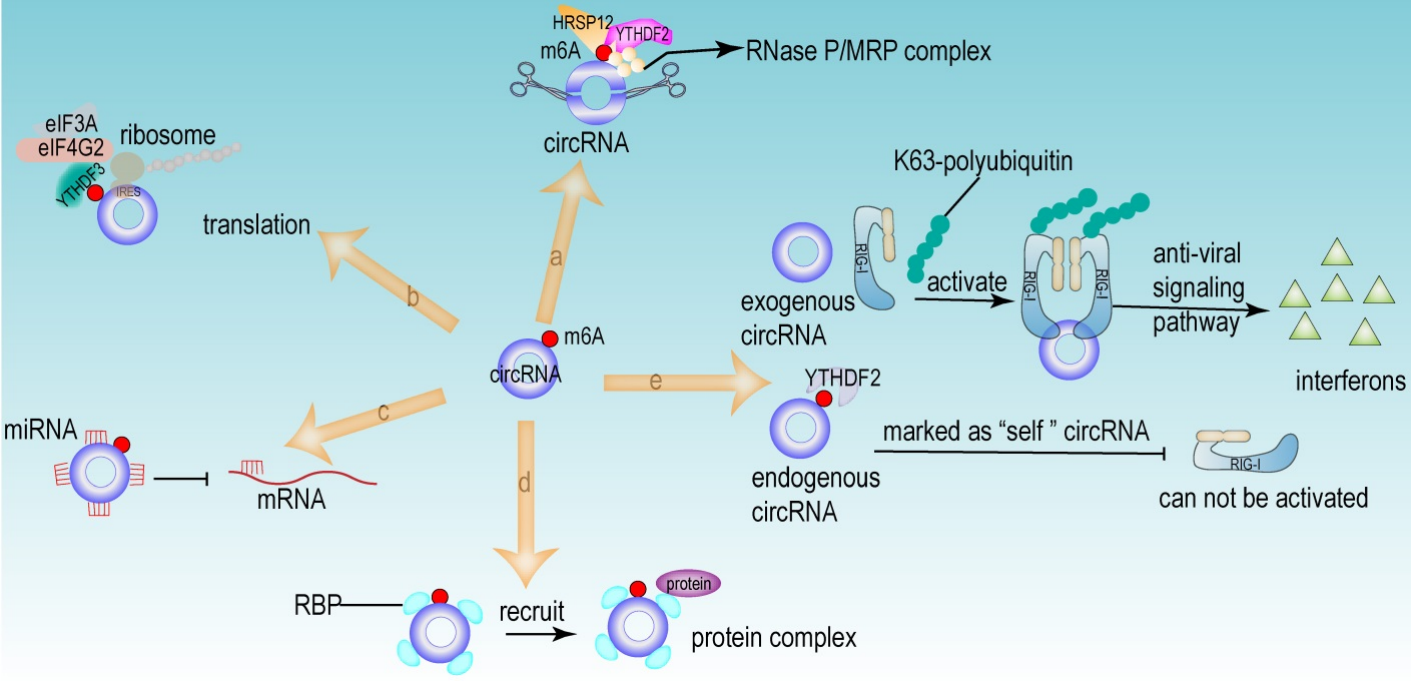
** The functions of circRNA modified by m6A.** (a) m6A regulated the stability of circRNA. The m6A modification site of circRNA could be recognized by “reader” YTHDF2. HRSP12 linked YTHDF2 to RNase P/MRP complex, therefore regulating circRNA degradation by YTHDF2-HRSP12-RNase P/MRP pathway. (b) m6A promoted the translation of circRNA in a cap-independent manner. m6A modification could be recognized by “reader” YTHDF3, which further recruited translation initiation factor (such as eIF4G2 and eIF3A) and ribosome to IRES for m6A-initiated circRNA translation process. (c) m6A facilitated circRNA to act as miRNA sponge. (d) m6A regulated the interaction between RBP and circRNA. (e) m6A recognized and participated the immunoreaction mediated by circRNA. Exogenous RNA (without m6A modification) activated RIG-I under the circumstance of K63-polyubiquitin, and then led to the activation of interferon production through anti-viral signaling pathway. While the endogenous circRNA (modified by m6A) could be recognized by YTHDF2 and then this endogenous circRNA was marked as “self” circRNA, thus blocking the activation of RIG-I.

**Figure 5:**
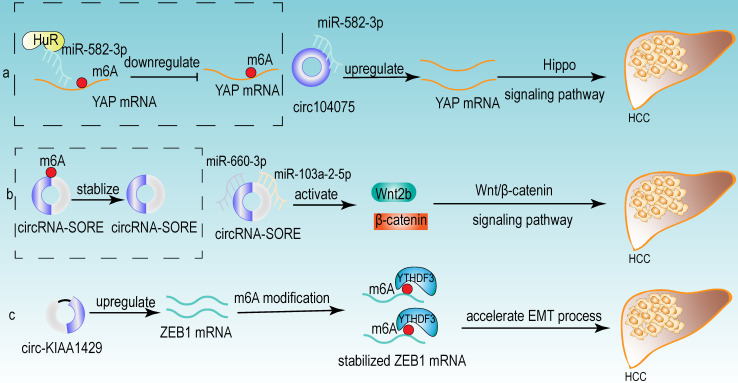
** Some examples of the cross-talk of circRNA and m6A modification in HCC.** (a) The m6A modification of YAP promoted the binding between miR-582-3p and the YAP mRNA. In addition, it promoted HuR binding and stabilizing miR-582-3p, so as to downregulate the expression of YAP. circ104075 could upregulate YAP expression by absorbing miR-582-3p, thus contributing to HCC progression through Hippo signaling pathway. (b) m6A modification stabilized circRNA-SORE, and circRNA-SORE acted as miR-103a-2-5p and miR-660-3p sponge so as to activate Wnt/β-catenin signaling and promote the progression of HCC. (c) ZEB1 acted as a target of circ-KIAA1429 and could be recognized by m6A “reader” YTHDF3, which could enhance the stability of ZEB1 mRNA. ZEB1 was also a critical transcription factor of EMT process. Therefore, upregulating the expression of ZEB1 by circ-KIAA1429 could accelerate the EMT process, and further facilitate the development of HCC.

From the above studies, we can conclude that both m6A modification and circRNA are closely associated with the development of HCC. Further researches revealed that circRNA can be modified by m6A modification and that m6A modification has an important function in maintaining the biological activities of circRNAs in the following perspectives: (1) m6A modification regulates the stability of circRNA (**Fig 4a**). m6A modification could be recognized by YTHDF2. HRSP12 linked YTHDF2 to RNase P/MRP, and regulated circRNA degradation by the YTHDF2-HRSP12-RNase P/MRP pathway 107, 108. (2) m6A modification promotes the translation of circRNA (**Fig 4b**). m6A modification promoted the translation of circRNA in a cap-independent manner. m6A modification could be recognized by “reader” YTHDF3, which further recruited translation initiation factor (such as eIF4G2 and eIF3A) and ribosome to IRES for m6A-initiated circRNA translation process 109. (3) m6A modification influences tumor progression by regulating circRNA-miRNA interactions (**Fig 4c**) 110. (4) m6A modification impacts the interactions between circRNA and RBPs (**Fig 4d**) 111. (5) m6A modification labels endogenous RNA to distinguish it from exogenous RNA, so as to avoid recognition and attack by the autoimmune system (**Fig 4e**). Exogenous RNA (without m6A modification) activated RIG-I under the circumstance of K63-polyubiquitin, and then led to the activation of interferon production through anti-viral signaling pathway. While endogenous circRNA modified by m6A could be recognized by YTHDF2 and marked as “self” circRNA, thus blocking the activation of RIG-I 112, and serving as a novel mechanism for immune escape of exogenous RNA viruses 113. In addition, the proportion of m6A modifications is closely related to the functions of circRNAs 114, 115. Therefore, the cross-talk of circRNA and m6A modification may play an even more important role in HCC development. For example, the m6A modification of YAP promoted the binding between miR-582-3p and the YAP mRNA. In addition, it promoted HuR binding and stabilizing miR-582-3p, so as to downregulate the expression of YAP. circ104075 could upregulate YAP expression by absorbing miR-582-3p, thus contributing to HCC progression through Hippo signaling pathway (**Fig 5a**) 116. circRNA-SORE promoted the development of HCC by acting as a miRNA sponge to segregate miR-103a-2-5p and miR-660-3p, then competitively activating Wnt/β-catenin signaling. Elevated m6A levels stabilized circRNA-SORE (**Fig 5b**) 117. ZEB1 (a critical transcription factor of EMT process) was a target gene of circ-KIAA1429. YTHDF3 could enhance the stability of ZEB1 mRNA in an m6A-dependent manner in order to promote the EMT process and facilitate the development of HCC (**Fig 5c**) 118.

m6A modification has an extremely important role in circRNAs, but few studies have combined the analysis of m6A modification and circRNAs or m6A-modified circRNAs in HCC. More extensive studies on the relationship among m6A modification, circRNA, and HCC carcinogenesis can be excavated to conduct further investigations and might become a new hot spot for HCC studies.

## Introduction to common databases for m6A modification and circRNA studies

### m6A modification databases

#### Databases based on MeRIP-seq

m6A regulation based on nucleotide sequences and with the development of secondary generation sequencing, the technique of MeRIP-seq for m6A sequencing has been widely used. For example, there are downstream analysis databases based on MeRIP-seq data, such as RNAmod, which provides information about the distribution of RNA modifications, modification coverage for different gene features, and functional annotation of modified mRNAs to annotate diverse kinds of mRNA modifications in different species 119.

RMBase v2.0 expands on the RMBase database with 600 datasets from 47 studies involving 13 species and 1,397,000 modification sites. The RMBase v2.0 database integrates epigenomic transcriptome sequencing data to explore RNA posttranscriptional modifications and their association with miRNA binding, disease-related single nucleotide polymorphisms (SNPs) and relationships with RBPs. In addition, the database includes predictions for m5c, m1A and others 120, 121.

The Met-DB v2.0 database predicts significantly more m6A peaks and single base loci in a specific context compared to the Met-DB database from 185 samples of 7 species from 26 independent studies. In addition, the Met-DB v2.0 database integrates a database of m6A "writers", "erasers" and "readers” and is a good tool for understanding m6A function 122, 123.

The WHISTLE database is a transcriptome-wide database for m6A RNA methylation site prediction. In addition to conventional sequence features, WHISTLE integrates 35 other genomic features for more accurate m6A site prediction. The WHISTLE database is a network-based approach to explore biological processes affected by changes in individual m6A sites by integrating RNA methylation profiles, gene expression profiles and protein-protein interaction data 124.

The REPIC database records approximately 10 million peaks from publicly available m6A-seq and MeRIP-seq data from 672 samples from 49 studies, covering 61 cell lines or tissues from 11 organisms. REPIC allows users to query m6A modification sites by specific cell lines or tissue types, which can present a comprehensive map of m6A modification sites, histone modification sites and related chromatin 125.

### Sequence analysis databases

The BERMP database, a novel cross-species identification tool, is based on deep learning, which can predict m6A regulatory sites with high confidence. The prediction accuracy is closely related to data size and compensated by the integration of a random forest classifier with a novel encoding of enhanced nucleic acid content. Excellent performance was achieved in both cross-validation tests and independent tests. The BERMP database performed well on the mammalian dataset but poorly on the yeast dataset 126.

The SRAMP database is a sequence-based m6A prediction database that combines three random forest classifiers that exploit their positional nucleotide sequence patterns, k-nearest neighbor information, and position-independent nucleotide pairing spectra features to depict the sequence context around m6A sites. SRAMP uses either genomic sequences or cDNA sequences as the input sequence and can achieve competitive performance in cross-validation tests and rigorous independent benchmark tests 127.

iMRM can simultaneously predict multiple loci for m6A, m5C, m1A, ψ and A-to-I modifications in Homo sapiens, Mus musculus and Saccharomyces cerevisiae. iMRM extracts optimally characterized modifications using feature selection techniques and is a good method for simultaneously identifying modifications occurring on different nucleotides 128.

### Other m6A modification databases

Most prediction models are based only on sequence features and use SVM or random forest as classification methods, while the HSM6AP database creatively weights the HSM6AP samples during the training process and is an m6A high-precision prediction database based on multiple weights and features. The HSM6AP database contributes to the effect of the fusion sequence and gene-level feature extraction on the accuracy of methylation site recognition 129.

m6Avar, which was derived from miCLIP/PA-m6A-seq experiments (high confidence), MeRIP-seq experiments (medium confidence) and transcriptome-wide predictions (low confidence), integrated the RBP-binding regions, miRNA targets and splicing sites associated with variants 130.

In addition, m6Acorr corrects the existing laboratory bias in RNA m6A methylation profiles and performs profile comparisons on the corrected datasets 131; M6A2Target provides a database of validated m6A targets and potential m6A targets 132, and the WITMSG database predicts m6A modification sites of introns 133 (**Table 3**).

### circRNA databases

circRNA databases are essential for transcriptomics studies. The CircAtlas database was developed based on 1070 RNA-seq samples collected from 19 normal tissues of 6 vertebrate species. It contains the expression patterns, conservation and functional annotation information of 1007087 highly reliable circRNAs 134.

The circRNADisease database is based on more than 800 papers reporting on circRNAs and diseases, with a collection of 354 pairs of interconnections between 330 circRNAs and 48 diseases to provide the circRNA expression pattern, experimental detection techniques, and circRNA-associated partners 135.

The MiOncoCirc database is the first circRNA database based on clinical tumor samples, containing circRNA expression information from more than 2000 clinical tumor samples, collecting the circRNA expression data of in situ and metastatic cancers, etc. 136.

exoRBase is a repository of circRNA, lncRNA and mRNA from human blood exosome RNA sequence analysis. exoRBase is characterized by the integration and visualization of RNA expression profiles of normal individuals and patients with different diseases based on standardized RNA-seq data and provides annotation, expression level and possible original tissues 137.

The CSCD database collected 228 RNA-seq samples from cancer and normal cell lines and identified 272152 cancer-specific circRNAs. The CSCD database can predict the miRNA response element sites and RBP sites of each circRNA and predict the potential open reading frames to facilitate the exploration of translatable circRNAs. Additionally, the CSCD database predicts the splicing events of each linear transcript and can also predict the splicing events of each circRNA, which can help us better understanding the connection between linear splicing and back-splicing 138.

circBase collects and integrates information on well-known circRNAs and newly discovered circRNAs from sequencing data, including human, mouse and other species, and is a fundamental database in circRNA research field 139.

The circInteractome database serves as the circRNA, miRNA and RBP public database and provides bioinformatics analysis of circRNA binding sites. It also predicts RBP and miRNA binding sites on human circRNAs and identifies potential circRNAs that can act as RBP sponges. Moreover, it could design primers to specifically detect circRNAs, design siRNAs for circRNA silencing, and identify potential IRESs 140 (**Table 4**).

In conclusion, several databases for m6A modification and circRNA studies have been developed and each of them contains different functions. We could reasonably select, integrate and apply the information from these databases according to the specific purpose of the research.

## m6A modifiers and circRNAs as biomarkers and therapeutic targets for HCC

The application of biomarkers is crucial in all stages of cancer and has become one of the main avenues for cancer diagnosis, prognosis and progression monitoring 141. A qualified biomarker should be sensitive, specific, reproducible, stable and clinically useful.

### m6A modifiers as HCC biomarkers

**Figure 6:**
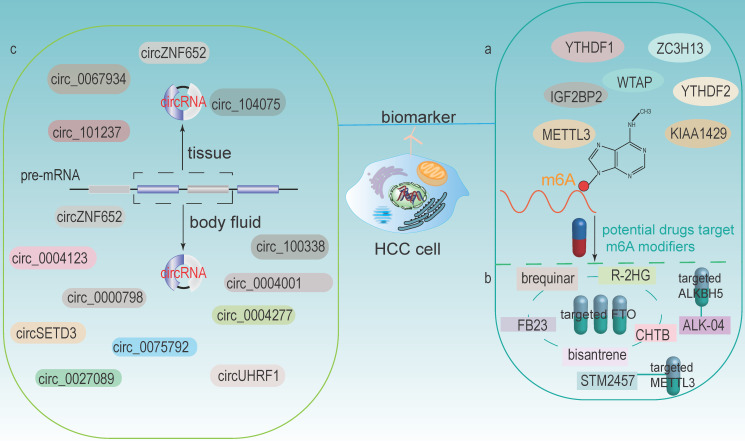
** m6A modifiers and circRNAs as potential biomarkers and therapeutic targets for HCC.** (a) m6A modifiers act as biomarkers in HCC. (b) Some drugs targets on m6A modifiers (including FTO, ALKBH5 and METTL3). (c) circRNAs in HCC tissues and body fluids could both be potential biomarkers.

m6A modification is the most abundant modification in eukaryotes and is closely associated with HCC development. The expression levels of m6A modifiers could be tested in liver tissue biopsy; thus, m6A modifiers are considered as potential biomarkers and prognostic indicators for HCC (**Fig 6a**). Qu et al. found that YTHDF1, YTHDF2, and KIAA1429 were significantly associated with the WHO stage of HCC and acted as independent prognostic markers for survival of HCC 42. In addition, METTL3 was identified as an independent prognostic factor for recurrence-free survival (RFS) of HCC 142. Chen et al. demonstrated that WTAP was highly expressed in HCC and its expression was an independent predictor of HCC patients' survival 27. YTHDF1 was significantly upregulated, exerted an important role in regulating cell cycle progression and metabolism in HCC. It also positively correlated with pathological stage. Therefore, it might be a potential new therapeutic and prognostic target for HCC 41. Higher expression of and IGF2BP2 in HCC tissues was associated with poor prognosis, which indicated another potential biomarker to predict HCC prognosis 38.

### m6A modifiers as potential therapeutic targets in cancers

In recent years, several small molecule inhibitors of m6A modifiers have been identified or developed, implying that m6A modifiers can be used as therapeutic drug targets and applied in the clinic (**Fig 6b**). Currently, the majority of m6A inhibitors discovered are targeting at FTO and ALKBH5. For example, Qiao et al. found that CHTB is an inhibitor of FTO, and this novel molecule provided a direction for further development of more selective and effective FTO inhibitors 143. Huang et al. identified that targeting FTO with two potential inhibitors, FB23 and FB23-2, have the potential to treat acute myelocytic leukemia (AML). These two inhibitors could directly bind to FTO and selectively inhibit the activity of FTO, thereby significantly inhibiting cell proliferation 144. Su et al. showed that bisantrene and brequinar (are also inhibitors of FTO), significantly attenuated the initiation of cellular self-renewal and reprogrammed leukemic stem cells by inhibiting the expression of the immune checkpoint gene LILRB4 145. Su et al. revealed that the tumor metabolite R-2HG could act as an inhibitor of FTO, leading to the increased methylation and decreased expression of c-MYC and CEBPA, thereby blocking proliferation, cell cycle and inducing apoptosis in AML and glioma cells 146. ALK-04, an inhibitor of ALKBH5, might serve as a potential therapeutic target to enhance immunotherapy outcomes in melanoma, colorectal cancer and other cancers 147. In addition, a recent research has showed that a small inhibitor STM2457 could target m6A “writer” METTL3 to reduce AML cells growth and increase its apoptosis 148.

The discovery of new m6A inhibitors has paved the way for m6A-based therapies; however, little is known about their roles in HCC. Moreover, the specificity and side effects of these drugs should be clarified to better promoting their application to HCC. In addition, determining whether these inhibitors affect other RNA modifications such as m1A is also of importance, as it could lead to uncertain consequences. Therefore, more in-depth studies of these inhibitors in vitro and in vivo are urgently requested 149. Besides, some m6A inhibitors can affect the immunotherapy of tumors, so combining appropriate m6A inhibitors with immune checkpoint blockade might achieve more efficient therapeutic effects.

### circRNAs as biomarkers for HCC

circRNA can be detected in tissue samples, plasma, serum, saliva, urine, latex, etc. And its expression profile appears to be cell-specific or stage-specific 150. Memczak et al. sequenced RNA in human peripheral blood and identified circRNA as an easily accessible potential biomarker in body fluids 151. Li et al. identified a large amount of exosomal RNAs in serum, which can be used as biomarkers for cancer diagnosis 152. These features make circRNAs be possible prospective biomarkers (**Fig 6c**).

### circRNAs in tissues

Numerous studies have now identified circRNAs in HCC tissues can be used as potential biomarkers. For example, patients with highly expressed circ-ZNF652 in HCC were more likely to suffer from vascular invasion, intrahepatic metastasis and distant metastasis, which were closely associated with a poor prognosis 153. circRHOT1 was a potential biomarker that repressed the progression of HCC by recruiting TIP60 to initiate NR2F6 expression 76. circ_0067934 stimulated invasion and metastasis of HCC, while circMTO1 negatively regulated the progression of HCC 154. Zhang et al. discovered that circ_104075 has potential as a new diagnostic biomarker for HCC, and using circ_104075 as a therapeutic target may provide a new strategy for the diagnosis and treatment of HCC 116. Moreover, the expression of circRNA_101237 was associated with tumor size, lymph node metastasis, and TNM stage. The serum circRNA_101237 level was an independent predictor of prognosis in HCC patients 102.

### circRNAs in body fluids

The role of circRNAs in body fluids as biomarkers of HCC has also been identified. For example, circUHRF1 was proven in plasma exosomes, and its high expression was associated with a decreased NK cell ratio. Therefore, it diminished NK cell infiltration and led to resistance of anti-PD1 immunotherapy, thus resulting in poor prognosis in HCC patients 98. circPTGR1 (circRNAs, hsa_circ_0008043, hsa_circ_0003731, and hsa_circ_0088030), a kind of circRNA with three subtypes that was upregulated in serum exosomes of HCC patients with metastasis-free or low metastatic capacities, they were closely related to clinical staging and prognosis 155. Besides, Sun et al. revealed that hsa_circ_0004001, hsa_circ_0004123, and hsa_circ_0075792 were upregulated in HCC blood samples and positively correlated with TNM stage and tumor size. Additionally, the combination use of these three biomarkers had much higher sensitivity and specificity in the diagnosis of HCC 156. High expression of circ_0000798 in peripheral blood was related to poor overall survival in HCC patients, and circ_0000798 had the potential as a diagnostic or predictive biomarker for HCC 157. Furthermore, circulating circSETD3 showed predictive ability for HCC microvascular invasion, and the expression level of circSETD3 was negatively correlated with HCC invasion and metastasis 158. Zhu et al. identified plasma hsa_circ_0027089 as a possible new biomarker for the diagnosis of hepatitis B-associated HCC via a clinical investigation 159. circ_0004277 was significantly upregulated in HCC cells, tissues and plasma exosomes. Exosomal circ_0004277 from HCC cells enhanced the circ_0004277 expression in surrounding normal cells through cellular communication, thus stimulating EMT process and promoting the invasion of HCC cells 160. In addition, the expression of exosomal circZNF652 was upregulated in the serum of HCC patients and was closely associated with the proliferation, migration, invasion and glycolysis of HCC cells, suggesting that it might become a novel biomarker for predicting the malignant characteristics of HCC 161. circ_100338 was highly expressed in metastatic HCC cells as well as exosomes, which could significantly affect tumor angiogenesis and might be a useful indicator of pulmonary metastasis and poor survival 87.

## Conclusions and perspectives

Many studies have shown that alterations of m6A modification and circRNA affect the development and progression of HCC, and they have important implications in the diagnosis, treatment and prognosis of HCC. In addition, circRNAs, which are highly abundant and stable, exist in a variety of extracellular fluids, including saliva, blood and urine, and can become ideal biomarkers and therapeutic targets for HCC 152, 162. circRNAs can be modified by m6A, which makes them more stable, so m6A modification might serves as an essential regulatory mechanism for the circRNAs to exert their functions. It is worthwhile to pay more attention to the interplay among m6A modification, circRNA, and HCC, because m6A modification as well as circRNA could significantly modulate the development of HCC, and the cross-talk of circRNA and m6A modification in HCC has been rarely studied nowadays.

Most of the current studies focused on the downstream targets of m6A modification, and further studies are needed to investigate the upstream mechanism of m6A modification. In terms of m6A modifiers as potential drug targets, some inhibitors have been investigated; however, most of these small molecule inhibitors target at FTO and ALKBH5, and their effects on HCC have not been extensively observed nowadays. Additionally, the inhibitors of other m6A regulators and their applications in HCC are needed to be investigated further. Moreover, the roles of m6A motif-associated SNPs in HCC have not been well studied, and these SNPs may alter the fate of target mRNAs, therefore leading to different consequences. Besides, the specificity of m6A modification detection has yet to be addressed; for example, antibodies used to recognize m6A modifications exhibit nonspecific binding and can also bind to another kind of RNA modification m6Am (N(6),2'-O-dimethyladenosine) 163. Although antibody-free assays have been introduced to detect m6A sites such as DART-SEQ (deamidation near RNA modification targets), they still have limitations such as low reproducibility, and need further improvement 164.

Stable expressions of circRNAs in HCC tissues, cells, exosomes and plasma provide new avenues for cancer diagnosis and treatment. However, the translational functions of circRNAs have been rarely studied and the current tools used to predict and identify their encoded proteins or peptides are still limited. In addition, Ma et al. pointed out that most circRNAs are named according to their host genes or functions, and when multiple circRNAs originate from the same host gene and have different functional roles, the naming situation is rather confused. Therefore, a novel standard circRNA naming rule is needed to construct in the related circRNA databases 95. Taken together, with the development of bioinformatics technology, the interaction networks of circRNA, m6A modification and HCC carcinogenesis will be more extensively studied. And more regulatory mechanisms and therapeutic targets that contribute to HCC development will be exploited.

## Figures and Tables

**Table 1:** The relationship between m6A modification and HCC

Category	m6A regulators	Up/down	Functions	Mechanisms	Refs
“writers”	METTL3	up	promote proliferation, migration and colony formation of HCC	NA	17
“writers”	METTL3	up	facilitate glycolysis	activate the mTORC1 pathway	18
“writers”	METTL3	up	promote the formation of vasculogenic mimicry	affect the translation of YAP1 mRNA	19
“writers”	METTL3	down	enhance the resistance to sorafenib	abolish METTL3-mediated FOXO3 mRNA stability	20
“writers”	METTL14	up	promote metastasis	upregulate m6A methylation of CSAD, GOT2, and SOCS2	23
“writers”	METTL14	down	inhibit migration, invasion and EMT	regulate EGFR/PI3K/AKT signaling pathway	24
“writers”	METTL14	up	enhance the resistance to sorafenib	reduce the expression of HNF3γ	25
“writers”	KIAA1429	up	promote proliferation and metastasis	Induce degradation of GATA3 precursor mRNA	26
“writers”	WTAP	up	boost HCC cell proliferation	the HuR-ETS1-p21/p27 axis	27
“writers”	ZC3H13	up	prognostic indicators for HCC	may associated with RAS-ERK pathway	28,29
“erasers”	FTO	up	poor prognosis	demethylation of PKM2 mRNA and speed up the translation	32
“erasers”	FTO	down	induce metastasis	downregulate m6A-modified GNAO1 and upregulated its mRNA expression	33
“erasers”	FTO	up	promote glucose metabolism	reduce CUL4A protein abundance	34
“erasers”	ALKBH5	down	promote proliferation and invasive	repress LYPD1	30,35
“readers”	YTHDC2	up	poor prognosis	small nucleotide polymorphisms	36
“readers”	IGF2BP1/IGF2BP3	up	poor prognosis	stabilize LINC01138 transcript	37
“readers”	IGF2BP2	up	promote HCC proliferation	enhance the stability of FEN1 mRNA	38
“readers”	HNRNPA2B1	down	inhibit HCC migration	decreased the stability of p52 and p65 mRNA	39
“readers”	NKAP	up	promote the proliferation and invasion	AKT signaling pathway	40
“readers”	YTHDF1	up	influence pathological stage	regulate cell cycle progression and metabolism	41
“readers”	YTHDF2	up	facilitate HCC stem cell phenotype and metastasis	increase OCT4 protein expression	42,43
“readers”	YTHDF2	down	inhibit the occurrence of HCC	accelerate the degradation of EGFR mRNA	44

**Table 2:** The relationship between circRNA and HCC

CircRNA	CircBase ID/common name	Gene symbol	Up/ down	Functions	Mechanisms	Refs
circRNA-SORE	circRNA_104797	NA	up	decrease cell killing ability of sorafenib	bound and stabilize YBX1	59
circRNA_0001955	circRNA_0001955	NA	up	promote tumorigenesis of HCC	adsorption of miR-516a-5p	75
circRHOT1	hsa_circRNA_102034	NA	up	promote HCC growth and metastasis.	increase the level of NR2F6	76
circRNA_0067934	circRNA_0067934	NA	up	boost proliferation, metastasis and invasion	activate FZD5/Wnt/β-catenin signaling pathway	77
circ_0000502	circ_0000502	NA	up	promote proliferation and invasion	mediate the expression of miR-124	78
hsa_circ_0051443	hsa_circ_0051443	TRAPPC6A	up	facilitate apoptosis and block the cell cycle	regulate extracellular communication in the tumor microenvironment	79
cSMARCA5	hsa_circ_0001445	SMARCA5	down	promote invasion	suppress the expression of tumor suppressor TIMP3	80
circASAP1	hsa_circ_0085616	ASAP1	up	promote proliferation and invasion	modulate miR-326/miR-532-5p-MAPK1 signaling pathway	81
circRNA-ADD3	hsa_circ_0020007	LOC100505933	up	suppress metastasis	downregulate the stability of EZH2	82
circMTO1	hsa_circRNA_0007874/ hsa_circRNA_104135	NA	down	promote proliferation and invasion	act as the sponge of oncogenic miR-9	83
circRNA_0003998	circRNA_0003998	NA	up	promote EMT	act as ceRNA for miR-143-3p and bind to PCBP1	84
circRNA Cdr1	hsa_circ_0001946	CDR1	up	promote proliferation and migration	adsorption of miR-1270	85
circRNA_104718	circRNA_104718	NA	up	promote proliferation, migration, invasion	regulate miR-218-5p/TXNDC5 signaling pathway	86
circRNA_100338	circRNA_100338	NA	up	promote metastasis	enhance invasiveness and angiogenesis	87
circRNA_5692	circRNA_5692	NA	down	promote invasion and metastasis	regulate the circRNA-5692-miR-328-5p-DAB2IP pathway	88
circGFRA1	NA	NA	up	promote angiogenesis	binding to miR-149	91
hsa_circ_0000092	hsa_circ_0000092	RPL5	up	promote invasion and angiogenesis	binding to miR-338-3p to upregulate the expression of HN1	92
circ_4911, circ_4302	circ_4911, circ_4302	NA	down	promote angiogenesis	promote the proliferation and migration of HUVECs	93
circMET	hsa_circ_0082002	MET	up	enhance immuno-suppression effect	modulate Snail/DPP4/CXCL10 axis	97
circUHRF1	NA	NA	up	immunosuppression	induce NK cell dysfunction	98
circMAT2B	hsa_circ_0074854	MAT2B	up	promote glycolysis	act as a miR-338-3p sponge to upregulate PKM2	99
circRNA_101505	circRNA_101505	NA	down	sensitize HCC cells to cisplatin	act as a sponge for miR-103 and stimulate the expression of NOR1	100
circRNA_102272	circRNA_102272	NA	up	facilitate cisplatin resistance	regulate the miR-326/RUNX2 axis	101
circRNA_101237	circRNA_101237	NA	up	promote cisplatin resistance	may increase DNA repair to alter cell accumulation	102
circFN1	hsa_circ_0058124	FN1	up	promote cisplatin resistance	act as miR-1205 sponge	103
circARNT2	hsa_circ_0104670	ARNT2	up	promote cisplatin resistance	regulate miR-155-5p/PDK1 pathway	104
circ_0003998	circ_0003998	NA	up	reduce sensitivity to doxorubicin of HCC cells	act as a ceRNA for miR-218-5p to regulate the expression of EIF5A2	105
circ_0003418	circ_0003418	NA	up	enhance the sensitivity of HCC cells to cisplatin	inhibit the Wnt/β-catenin pathway	106

**Table 3:** The summary of m6A modification databases

Database	Websites	Mechanisms	Functions	Refs
RNAmod	https://bioinformatics.sc.cn/RNAmod	based on MeRIP-seq	annotate diverse kinds of mRNA modifications in different species	119
RMBase v2.0	http://rna.sysu.edu.cn/rmbase	based on MeRIP-seq	explore RNA post-transcriptional modifications and their association with miRNA binding, disease-related SNPs and relationships with RBPs.	120, 121
Met-DB v2.0	http://compgenomics.utsa.edu/MeTDB/	based on MeRIP-seq	integrate a database of m6A "writers", "erasers" and "readers”	122, 123
WHISTLE	http://whistle-epitranscriptome.com	based on MeRIP-seq	explore biological processes affected by changes in individual m6A sites by integrating RNA methylation profiles, gene expression profiles and protein-protein interaction data	124
REPIC	https://repicmod.uchicago.edu/repic	based on MeRIP-seq	query m6A modification sites by specific cell lines or tissue types	125
BERMP	http://www.bioinfogo.org/bermp	based on sequence analysis	a cross-species identification tool	126
SRAMP	http://www.cuilab.cn/sramp/	based on sequence analysis	depict the sequence context around m6A sites	127
iMRM	http://www.bioml.cn/XG_iRNA/home	based on sequence analysis	simultaneously predict multiple loci for m6A, m5C, m1A, ψ and A-to-I modifications in Homo sapiens, Mus musculus and Saccharomyces cerevisiae	128
HSM6AP	http://lab.malab.cn/~lijing/HSM6AP.html	multiple weights and features	explore the effect of the fusion sequence and gene-level feature extraction on the accuracy of methylation site recognition	129
m6AVar	http://m6avar.renlab.org	based on miCLIP/PA-m6A-seq, MeRIP-seq and transcriptome-wide predictions	integrate the RBP-binding regions, miRNA-targets and splicing sites associated with variants	130
m6Acorr	http://www.rnanut.net/m6Acorr	based on normalizing RNA-seq and microarray data	correct laboratory bias in RNA m6A methylation profiles and perform profile comparisons on the corrected datasets	131
M6A2Target	http://m6a2target.canceromics.org	collect all reported m6A WERs	provide database of validated m6A targets and potential m6A targets	132
WITMSG	http://rnamd.com/intron/	based on sequence features and a variety of genomic characteristics	predict all possible intronic m6A sites	133
Database	Websites	Mechanisms	Functions	Refs
RNAmod	https://bioinformatics.sc.cn/RNAmod	based on MeRIP-seq	annotate diverse kinds of mRNA modifications in different species	119
RMBase v2.0	http://rna.sysu.edu.cn/rmbase	based on MeRIP-seq	explore RNA post-transcriptional modifications and their association with miRNA binding, disease-related SNPs and relationships with RBPs.	120, 121
Met-DB v2.0	http://compgenomics.utsa.edu/MeTDB/	based on MeRIP-seq	integrate a database of m6A "writers", "erasers" and "readers”	122, 123
WHISTLE	http://whistle-epitranscriptome.com	based on MeRIP-seq	explore biological processes affected by changes in individual m6A sites by integrating RNA methylation profiles, gene expression profiles and protein-protein interaction data	124
REPIC	https://repicmod.uchicago.edu/repic	based on MeRIP-seq	query m6A modification sites by specific cell lines or tissue types	125
BERMP	http://www.bioinfogo.org/bermp	based on sequence analysis	a cross-species identification tool	126
SRAMP	http://www.cuilab.cn/sramp/	based on sequence analysis	depict the sequence context around m6A sites	127
iMRM	http://www.bioml.cn/XG_iRNA/home	based on sequence analysis	simultaneously predict multiple loci for m6A, m5C, m1A, ψ and A-to-I modifications in Homo sapiens, Mus musculus and Saccharomyces cerevisiae	128
HSM6AP	http://lab.malab.cn/~lijing/HSM6AP.html	multiple weights and features	explore the effect of the fusion sequence and gene-level feature extraction on the accuracy of methylation site recognition	129
m6AVar	http://m6avar.renlab.org	based on miCLIP/PA-m6A-seq, MeRIP-seq and transcriptome-wide predictions	integrate the RBP-binding regions, miRNA-targets and splicing sites associated with variants	130
m6Acorr	http://www.rnanut.net/m6Acorr	based on normalizing RNA-seq and microarray data	correct laboratory bias in RNA m6A methylation profiles and perform profile comparisons on the corrected datasets	131
M6A2Target	http://m6a2target.canceromics.org	collect all reported m6A WERs	provide database of validated m6A targets and potential m6A targets	132
WITMSG	http://rnamd.com/intron/	based on sequence features and a variety of genomic characteristics	predict all possible intronic m6A sites	133

**Table 4:** The summary of circRNA databases

Database	Websites	Features	Functions	Refs
circAtlas	http://circatlas.biols.ac.cn/	contain 1007087 highly reliable circRNA	provide the expression patterns, conservation and functional annotation information	134
circRNADisease	http://cgga.org.cn:9091/circRNADisease/	based on the collection of 354 pairs of interconnections between 330 circRNAs and 48 diseases	provide the circRNA expression pattern, experimental detection techniques, circRNA-associated partners, a brief description of circRNA biological function	135
MiOncoCirc	https://mioncocirc.github.io/	based on more than 2000 clinical tumor samples	provide the circRNA expression data of in-situ and metastatic cancers	136
exoRBase	http://www.exoRBase.org	a repository of circRNA, lncRNA and mRNA from human blood exosome RNA sequence analysis	integrate and visualize RNA expression profiles of normal individuals and patients based on RNA-seq data	137
CSCD	http://gb.whu.edu.cn/CSCD	collect 228 RNA-seq samples and identify 272152 cancer-specific circRNAs	predict the miRNA response element sites and RBP sites and the potential open reading frames to facilitate the exploration of translatable circRNAs	138
circBase	http://www.circbase.org/	merge circRNAs and downloaded their expression profile	provide scripts to identify known and novel circRNAs in sequencing data	139
CircInteractome	http://circinteractome.nia.nih.gov	search the circRNA, miRNA and RBP public database	predict circRNA binding sites, RBP and miRNA binding sites	140

## Abbreviation

3'-UTR: 3'-untranslated region
AML: acute myelocytic leukemia
AR: androgen receptor
CAEs: cancer-associated endothelial cells
CAFs: cancer-associated fibroblasts
CAR-T: chimeric antigen receptor engineered T-cell immunotherapy
ciRNAs: intronic circRNAs
ECM: extracellular matrix
ecRNAs: exonic circRNAs
eIciRNAs: exon-intron circRNAs
EMT: epithelial-mesenchymal transition
FLT3: FMS-like tyrosine kinase-3
FTO: fat mass and obesity-associated protein
GNAO1: guanine nucleotide-binding protein G (o) subunit alpha
HCC: hepatocellular carcinoma
HNF3γ: hepatocyte nuclear factor 3γ
HNRNP: heterogeneous nuclear ribonucleoprotein
HUVECs: human umbilical vein endothelial cells
IRES: internal ribosome entry site
LYPD1: LY6/PLAUR domain 1
m6A: N6-methyladenosine
mTORC1: mTOR complex 1
NKAP: NF-κβ-associated protein
NOR1: oxidored-nitro domain-containing protein 1
PTBP1: polypyrimidine binding protein 1
QKI: Quaking
RBP: RNA-binding protein
RFS: recurrence-free survival
SAM: S-adenosylmethionine
SNPs: single nucleotide polymorphisms
TACE: trans-arterial chemoembolization
TME: tumor microenvironment
VM: vasculogenesis mimicry
